# Determinants of the heart rate variability in type 1 diabetes mellitus

**DOI:** 10.3389/fendo.2023.1247054

**Published:** 2023-10-03

**Authors:** Máté Hajdu, Konstandia Garmpis, Vivien Vértes, Noémi Vorobcsuk-Varga, Gergő Attila Molnár, László Hejjel, István Wittmann, Réka Faludi

**Affiliations:** ^1^ Heart Institute, Medical School, University of Pécs, Pécs, Hungary; ^2^ Medicover Clinic, Pécs, Hungary; ^3^ 2nd Department of Internal Medicine and Nephrological Center, Medical School, University of Pécs, Pécs, Hungary

**Keywords:** type 1 diabetes mellitus, heart rate variability, cardiac autonomic neuropathy, glycemic control, cardiovascular risk factor

## Abstract

**Background:**

Evaluation of heart rate variability (HRV) detects the early subclinical alterations of the autonomic nervous system. Thus, impaired HRV is the earliest subclinical marker of cardiac autonomic neuropathy (CAN) in type 1 diabetes mellitus (T1DM).

**Objectives:**

We aimed to explore the HRV parameters in asymptomatic T1DM patients and compare them with the results obtained in healthy subjects. Potential associations between HRV parameters and the established risk factors for CAN and cardiovascular diseases were also investigated.

**Methods:**

Seventy T1DM patients (38 ± 12 years, 46 females) and 30 healthy subjects were enrolled into the study. For HRV analysis, beat-to-beat heart rate was recorded for 30 min. The less noisy 5-min segment of the recording was analyzed by Bittium Cardiac Navigator HRV analysis software. Time domain, frequency domain, and nonlinear indices were calculated.

**Results:**

Regarding ratio of low to high frequency component (LF/HF), no differences were found between the two populations (*p* = 0.227). All the further, time domain, frequency domain, and nonlinear HRV indices were significantly lower in T1DM patients (each *p* < 0.001). In multiple linear models, disease duration remained the only independent predictor of LF/HF ratio (*p* = 0.019). HbA_1c_ was found to be significant independent predictor of all further time domain (SDNN, *p* < 0.001; rMSSD, *p* < 0.001), frequency domain (VLF, *p* < 0.001; LF, *p* = 0.002; HF, *p* = 0.006; Total Power, *p* = 0.002), and nonlinear indices (SD1, *p* = 0.006; SD2, *p* = 0.007), alone, or in combination with other factors, such as age or body mass index.

**Conclusion:**

Asymptomatic T1DM patients have significantly reduced overall HRV as compared with healthy subjects, indicating subclinical CAN. Quality of the glycemic control is important determinant of HRV among T1DM patients. This relationship is independent of other risk factors for CAN or cardiovascular diseases.

## Introduction

Type 1 diabetes mellitus (T1DM) is one of the most common chronic conditions, which affect the young adult population. Various micro- and macrovascular complications are frequent in this disease, resulting in at least 10-fold increase in cardiovascular morbidities as compared with age-matched healthy persons (1, 2). Cardiac autonomic neuropathy (CAN) is a consequence of the diabetes and is defined as the impairment of the cardiovascular autonomic control (3). It is one of the most neglected long-term complications of diabetes, remaining subclinical until late stages of the disease (4). Diabetic patients with CAN have a 3.4 times higher risk of mortality than patients without CAN (5). It has been proved that, in T1DM, major risk factors for CAN are age, duration of diabetes, glycemic control, systemic hypertension, dyslipidemia, obesity, smoking habits, and the existence of diabetic microvascular complications (nephropathy or microalbuminuria and retinopathy) (6–9).

Evaluation of heart rate variability (HRV) detects the early subclinical alterations of the autonomic nervous system. Thus, impaired HRV is the earliest subclinical marker of CAN in asymptomatic patients with T1DM (3, 4, 10, 11). Reduced HRV has already been reported in T1DM patients (12–15). In addition, it has been proved that major risk factors for CAN are significant determinants of the reduced HRV in this disease (16–19). These data, however, are limited and controversial.

Thus, we aimed to explore the HRV parameters in asymptomatic T1DM patients and compare them with the results obtained in healthy subjects. Potential correlations between HRV indices and the established risk factors for CAN and cardiovascular diseases were also investigated.

## Materials and methods

### Study population

Seventy-five asymptomatic T1DM patients without known cardiovascular disease were enrolled into our prospective investigation. Blood sampling for serum glycated hemoglobin (HbA_1c_%) and for other laboratory markers was performed within a 30-day period before enrollment. Thorough medical history was collected. Echocardiographic data were obtained by Philips Epiq 7 ultrasound system (Philips Healthcare, Best, the Netherlands). Treadmill stress test was performed using Bruce protocol. The exclusion criteria were as follows: atrial fibrillation, known diseases of the coronary— Coronary artery disease (CAD), or peripheral arteries, clinical diabetic nephropathy [macroalbuminuria (≥ 300 mg/day) and/or eGFR (estimated glomerular filtration rate) < 60 ml/min/1.73 m^2^] or retinopathy, impaired left ventricular systolic function (ejection fraction < 55%), significant valvular heart disease, echocardiographic suspicion of primary cardiomyopathies, abnormal treadmill stress test result indicative of CAD. Smokers, patients treated with angiotensin-converting enzyme (ACE) inhibitors/angiotensin receptor blockers (ARBs) as part of the standard care of diabetic nephropathy, or patients with well-controlled hypertension were not excluded.

Thirty age- and gender-matched healthy individuals without diabetes and any signs or symptoms of cardiovascular diseases were recruited as control population. Echocardiography and treadmill stress test were performed accordingly. The exclusion criteria were as follows: atrial fibrillation, impaired left ventricular systolic function (ejection fraction < 55%), significant valvular heart disease, echocardiographic suspicion of hypertension or primary cardiomyopathies, and abnormal treadmill stress test result indicative of CAD. Former or current smokers and volunteers with known dyslipidemia were not excluded from the study.

Subjects who have never smoked or who have smoked less than 100 cigarettes during their life were interpreted as non-smokers. Former smokers: subjects who have smoked at least 100 cigarettes in their lifetime but who had quit smoking at the time of the inclusion (quitted > 30 days ago). Current smokers were defined as having smoked at least 100 cigarettes during their life and still smoke cigarettes (20).

The study complied with the Declaration of Helsinki. The protocol was approved by the institutional ethics committee. Written informed consent was given by all participants before study inclusion.

### Assessment of HRV

Participants were asked to abstain from heavy exercise, alcohol, or caffeinated drinks within a day prior to the visit. Data were collected on standard room temperature, in the early afternoon period, minimizing the impact of the circadian rhythm. Participants were placed on a table in supine position, and requested to breathe at a normal pace, remain at rest, but awake and avoid conversation. Following a 15-min orthostatic adaptation period, beat-to-beat electrocardiogram (ECG) was recorded by Bittium Faros 360 ECG sensor (Bittium Corporation, Oulu, Finland) for 30 min. Records were visually inspected to avoid premature beats and artifacts. HRV parameters were obtained from the less noisy 5-min segment of the ECG recording by Bittium Cardiac Navigator HRV analysis software (Bittium Corporation, Oulu, Finland). Only samples with more than 95% of sinus beats were evaluated.

Time-domain indices were calculated including SDNN (standard deviation of the normal–normal intervals) and rMSSD (root mean square differences of successive normal–normal intervals).

Standard frequency-domain indices were computed by fast Fourier transformation: very low-frequency component or VLF (< 0.04 Hz), low-frequency component or LF (0.04 Hz–0.15 Hz), and high-frequency component or HF (0.15 Hz–0.4 Hz). In addition, the Total Power of the spectrum and the ratio of low to high frequency component (LF/HF) were calculated.

Nonlinear indices were provided by Poincaré plot analysis. Poincaré plot represents the RR_i+1_ interval as a function of the previous RR_i_ interval. SD1 and SD2 reflect the dispersion along minor and major axis of the fitted ellipse, respectively (21, 22).

### Statistical analysis

Categorical data were expressed as *n* (%). The frequencies of categorical parameters were compared by chi-square test. Normality of data was tested using Shapiro–Wilk test. Continuous variables with Gaussian distribution were reported as mean ± standard deviation (SD), whereas data whose normality was not confirmed were presented as median [interquartile range (IQR)]. Between-group comparisons were performed by independent samples Student’s t-test for normally distributed variables or by Mann-Whitney U test for variables not normally distributed.

Since HRV parameters did not display normal distribution, logarithmic transformation (ln) was implemented before applying linear regression analysis. Univariate predictors of the HRV parameters were determined by linear regression analysis. In a second step, multiple stepwise linear regression analysis was used. Variables with *p* < 0.1 on univariate analysis were incorporated into the multiple models. Variance inflation factor values > 2.5 were recognized indicative of collinearity. Partial regression plots (which displays the residuals of each variable after adjusting for the other variables in the multiple model) were used to visualize the correlations between risk factors and HRV indices. Partial correlation coefficients were reported on the plots.

*p* < 0.05 was considered significant. Statistical analysis was completed using IBM SPSS Statistics 27 software (IBM Corporation, Armonk, NY, USA).

## Results

Of a total of 75 patients, 70 (38 ± 12 years, 46 females) were eligible for the study. In four individuals, echocardiography was not feasible, due to poor acoustic windows. In one patient, significant ST segment changes detected during stress test were indicative of CAD. Subsequently, this diagnosis was verified by coronary angiography. HRV analysis was feasible in all patients.

Echocardiographic data of the study population have already been reported (23). Detailed clinical and laboratory characteristics of the T1DM patients are exhibited in **Table 1**. Altogether, 20 subjects were treated with antihypertensive drugs.

**Table 1:** Clinical data of the T1DM population and comparison with healthy subjects.

		T1DM patients (*n* = 70)	Healthy volunteers (*n* = 30)	
		Mean ± SD	Mean ± SD	*p*
Clinical characteristics				
Age (years)		38 (20.0) ^†^	34 (14.3) ^†^	0.709
Female gender *n* (%)		46 (66)	17 (57)	0.390
Body surface area (m^2^)		1.84 ± 0.2	1.88 ± 0.2	0.408
Body mass index (kg/m^2^)		23.5 ± 3.6	24.8 ± 4.2	0.152
Resting heart rate (beat/min)		73.7 ± 12.0	68.9 ± 7.5	**0.019**
Office blood pressure, systolic (mmHg)		135.8 ± 18.1	134.0 ± 14.7	0.630
Office blood pressure, diastolic (mmHg)		79.8 ± 9.8	78.7 ± 8.8	0.538
Disease duration (years)		21.0 ± 10.3		
Daily dose of insulin (U/kg)		0.64 ± 0.22		
Smoking	Never smoked n (%)	43 (61.4)	22 (73.3)	0.505
	Former smokers n (%)	13 (18.6)	3 (10)	
	Current smokers n (%)	14 (20)	5 (16.7)	
Laboratory data				
Current HbA1c (%)		7.6 ± 1.3		
Fasting glucose (mmol/l)		8.4 ± 4.6		
Fructosamine (μmol/l)		387.7 ± 65.3		
Creatinine (μmol/l)		73.6 ± 11.7		
eGFR (ml/min/1.73 m^2^)		95.9 ± 19.5		
Hemoglobin (g/l)		138.7 ± 15.2		
Total cholesterol (mmol/l)		4.7 ± 1.2		
Triglyceride (mmol/l)		1.0 ± 0.6		
Erythrocyte sedimentation rate (mm/h)		7.9 ± 7.4		
C-reactive protein (mg/l)		3.1 ± 4.1		
Medication				
ACE inhibitors/ARBs *n* (%)		17 (24)		
Calcium channel blocker *n* (%)		4 (6)		
Beta receptor antagonists *n* (%)		12 (17)		

Statistically significant p-values (p < 0.05) are formatted in bold. ^†^Reported as median (IQR).

### Comparison with healthy subjects

Patients and volunteers were matched for age, gender, body surface area, body mass index (BMI), office blood pressure, and smoking habits. Resting heart rate was significantly higher in T1DM patients compared to healthy individuals. This discrepancy, however, was clinically not remarkable.

**Figure 1:**
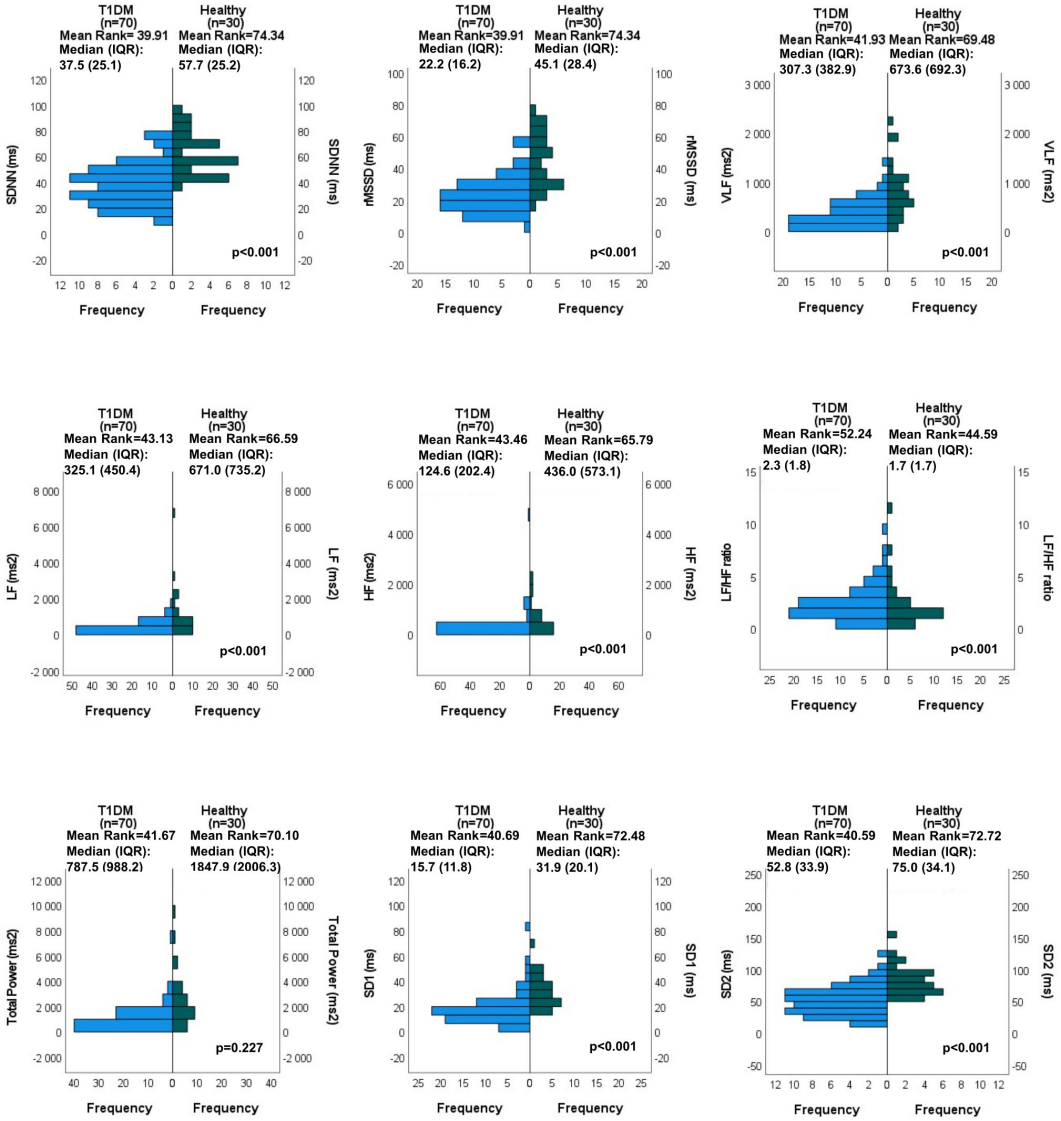
HRV parameters in the T1DM population and comparison with healthy subjects: Independent samples Mann-Whitney U test results.

HRV parameters of the T1DM patients and their comparison with those in healthy persons are displayed in **Figure 1**. Regarding LF/HF ratio, no differences were found between the two populations. Further time domain, frequency-domain, and nonlinear parameters of the HRV, however, were significantly lower in T1DM patients.

### Correlations between HRV parameters and the major risk factors for CAN

In univariate analyses, age, BMI, disease duration, systolic blood pressure, smoking, current HbA1c, eGFR, use of ACE inhibitors/ARBs, and use of beta receptor antagonists showed significant correlations with various HRV parameters. Correlations with borderline significance were found between gender and HRV parameters, whereas diastolic blood pressure, total cholesterol or triglyceride levels and use of calcium channel blockers did not show any significant correlation with them (**Table 2**).

**Table 2:** Univariate predictors of the HRV parameters in the T1DM population.

		Age (years)	Gender (male/female)	BMI (kg/m^2^)	Disease duration (years)	Office blood pressure, systolic (mmHg)	Office blood pressure, diastolic (mmHg)	Smoking (never/ previously/currently)	Current HbA1c (%)	eGFR (ml/min/1.73 m^2^)	Total cholesterol (mmol/l)	Triglyce-ride (mmol/l)	Use of ACE inhibitors/ARBs (yes/no)	Use of beta receptor antagonists (yes/no)	Use of calcium channel blockers (yes/no)
**ln SDNN**	r p	-0.365 **0.002**	-0.145 0.231	-0.356 **0.002**	-0.205 *0.094*	-0.240 **0.045**	-0.069 0.572	-0.260 **0.030**	-0.448 **< 0.001**	0.335 **0.007**	-0.088 0.526	-0.048 0.729	-0.230 *0.056*	-0.222 *0.065*	0.039 0.746
**ln rMSSD**	r p	-0.321 **0.007**	-0.101 0.407	-0.319 **0.007**	-0.218 *0.074*	-0.210 *0.081*	-0.135 0.265	-0.173 0.152	-0.409 **< 0.001**	0.345 **0.005**	-0.097 0.486	-0.015 0.914	-0.241 **0.044**	-0.185 0.126	0.002 0.986
**ln VLF**	r p	-0.318 **0.012**	-0.175 0.175	-0.250 *0.050*	-0.124 0.338	-0.229 *0.074*	-0.101 0.407	-0.198 0.123	-0.425 **< 0.001**	0.229 *0.073*	-0.063 0.650	-0.103 0.460	-0.220 *0.067*	-0.176 0.144	0.021 0.866
**ln LF**	r p	-0.438 **< 0.001**	-0.224 *0.062*	-0.380 **0.001**	-0.225 *0.065*	-0.272 **0.023**	-0.135 0.264	-0.222 *0.065*	-0.396 **< 0.001**	0.279 **0.025**	-0.129 0.353	-0.111 0.424	-0.333 **0.005**	-0.308 **0.009**	-0.103 0.396
**ln HF**	r p	-0.411 **< 0.001**	-0.086 0.478	-0.260 **0.030**	-0.330 **0.006**	-0.208 *0.083*	-0.094 0.440	-0.199 *0.098*	-0.387 **0.001**	0.301 **0.015**	-0.130 0.349	0.012 0.933	-0.234 *0.051*	-0.160 0.185	-0.047 0.702
**ln LF/HF ratio**	r p	0.088 0.469	-0.201 *0.096*	-0.114 0.347	0.284 **0.019**	-0.039 0.751	-0.039 0.751	0.027 0.823	0.109 0.371	-0.141 0.268	0.048 0.730	-0.199 0.149	-0.047 0.702	-0.193 0.109	-0.079 0.518
**ln Total Power**	r p	-0.405 **< 0.001**	-0.159 0.189	-0.300 **0.012**	-0.217 *0.076*	-0.275 **0.021**	-0.120 0.321	-0.249 **0.038**	-0.388 **< 0.001**	0.274 **0.028**	-0.122 0.379	-0.084 0.544	-0.263 **0.028**	-0.212 *0.078*	-0.041 0.734
**ln SD1**	r p	-0.352 **0.003**	-0.074 0.544	-0.307 **0.010**	-0.217 *0.075*	-0.216 *0.072*	-0.150 0.214	-0.172 0.154	-0.385 **0.001**	0.334 0.**007**	-0.123 0.375	-0.020 0.886	-0.250 **0.037**	-,193 0.110	-,013 0.916
**ln SD2**	r p	-0.385 **0.001**	-0.164 0.175	-0.369 **0.002**	-0.207 *0.091*	-0.234 *0.051*	-0.071 0.560	-0.288 **0.016**	-0.393 **< 0.001**	0.331 **0.007**	-0.106 0.447	-0.055 0.693	-0.240 **0.045**	-0.233 *0.052*	0.037 0.761

Statistically significant p-values (p < 0.05) are formatted in bold. 0.05 ≤ p < 0.1 values are formatted in italics.

**Figure 2:**
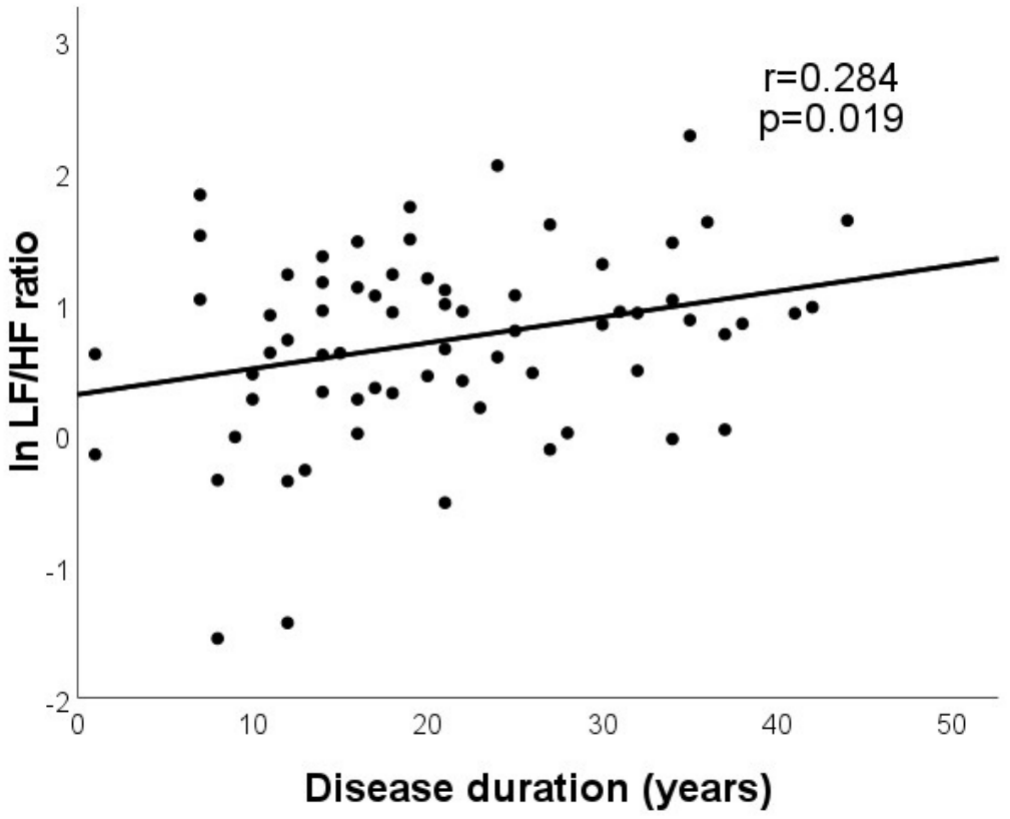
Disease duration was found to be the only independent predictor of LF/HF ratio in multiple model.

**Figure 3:**
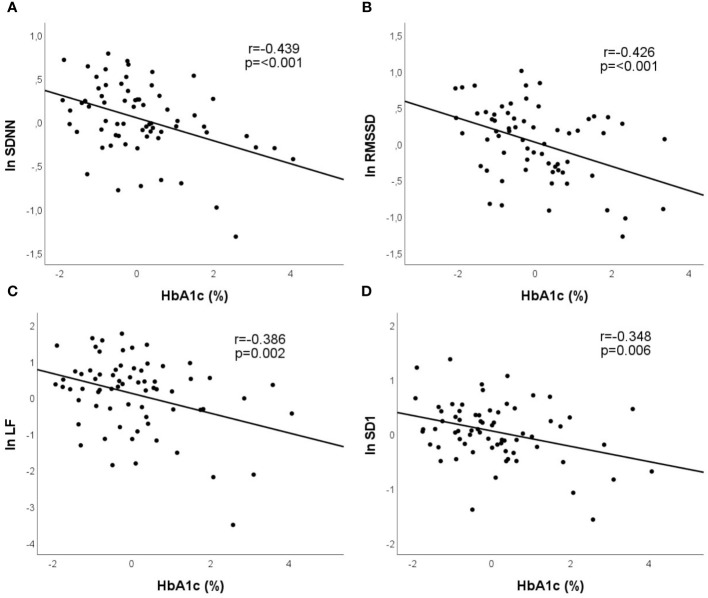
Partial regression plots demonstrate that in multiple models HbA_1c_ (%) shows significant correlation with SDNN **(A)**, rMSSD **(B)**, LF **(C)**, and with SD1 **(D)**. Partial correlation coefficients are reported.

In multiple linear model, disease duration remained the only independent predictor of LF/HF ratio (**Figure 2**). HbA_1c_, on the other hand, was proved to be the significant independent predictor of all the further time domain, frequency domain, and nonlinear indices, alone, or in combination with other factors, such as age or BMI (**Table 3**). Partial regression plots indicate that HbA_1c_ level correlates significantly with various HRV parameters in multiple models (**Figure 3**).

**Table 3:** Significant independent predictors of the HRV parameters in T1DM population: multivariate regression analyses.

	*B*	β	*p*	*F*	adj. *R* ^2^	*p*
ln SDNN				12.719	0.366	< 0.001
**HbA1c (%)**	-0.142	0.395	**< 0.001**			
**BMI (kg/m^2^)**	-0.027	-0.259	**0.016**			
**Age (years)**	-0.011	-0.257	**0.019**			
Disease duration (years)		-0.17	0.889			
Office blood pressure, systolic (mmHg)		-0.19	0.862			
Smoking (n/p/c)		-0.180	0.083			
eGFR (ml/min/1.73 m**^2^**)		0.119	0.308			
Use of ACE inhibitors/ARBs (y/n)		0.113	0.365			
Use of beta receptor antagonists (y/n)		0.085	0.472			
ln rMSSD				11.436	0.255	< 0.001
**HbA1c (%)**	-0.167	-0.405	**< 0.001**			
**eGFR (ml/min/1.73 m^2^)**	0.008	0.280	**0.015**			
Age (years)		-0.154	0.222			
BMI (kg/m**^2^**)		-0.215	0.060			
Disease duration (years)		-0.097	0.409			
Office blood pressure, systolic (mmHg)		-0.065	0.563			
Use of ACE inhibitors/ARBs (y/n)		-0.081	0.498			
ln VLF				14.108	0.172	< 0.001
**HbA1c (%)**	-0.321	-0.431	**< 0.001**			
Age (years)		-0.231	0.051			
BMI (kg/m**^2^**)		-0.185	0.112			
Office blood pressure, systolic (mmHg)		-0.216	0.061			
eGFR (ml/min**/**1.73 m**^2^**)		0.170	0.145			
Use of ACE inhibitors/ARBs (y/n)		-0.106	0.385			
ln LF				13.207	0.375	< 0.001
**HbA1c (%)**	-0.311	-0.336	**0.002**			
**Age (years)**	-0.034	-0.325	**0.003**			
**BMI (kg/m^2^)**	-0.072	-0.270	**0.012**			
Gender (male**/**female)		-0.185	0.071			
Disease duration (years)		-0.033	0.781			
Office blood pressure, systolic (mmHg)		-0.057	0.592			
Smoking (n/p/c)		-0.113	0.278			
eGFR (ml/min**/**1.73 m**^2^**)		0.012	0.917			
Use of ACE inhibitors/ARBs (y/n)		0.010	0.938			
Use of beta receptor antagonists (y/n)		0.011	0.927			
ln HF				11.975	0.265	< 0.001
**Age (years)**	-0.045	-0.353	**0.003**			
**HbA1c (%)**	-0.363	-0.326	**0.006**			
BMI (kg/m**^2^**)		-0.173	0.125			
Disease duration (years)		-0.111	0.388			
Office blood pressure, systolic (mmHg)		-0.088	0.434			
Smoking (n/p/c)		-0.102	0.364			
eGFR (ml/min**/**1.73 m**^2^**)		0.118	0.337			
Use of ACE inhibitors/ARBs (y/n)		-0.036	0.765			
ln LF/HF ratio				5.787	0.067	0.019
**Disease duration (years)**	0.020	0.284	**0.019**			
Gender (male**/**female)		-0.213	0.072			
ln Total Power				12.346	0.271	< 0.001
**HbA1c (%)**	-0.295	-0.357	**0.002**			
**Age (years)**	-0.031	-0.329	**0.005**			
BMI (kg/m**^2^**)		-0.196	0.079			
Disease duration (years)		0.004	0.978			
Office blood pressure, systolic (mmHg)		-0.133	0.232			
Smoking (n/p/c)		-0.122	0.271			
eGFR (ml/min**/**1.73 m**^2^**)		0.081	0.507			
Use of ACE inhibitors/ARBs (y/n)		-0.046	0.703			
Use of beta receptor antagonists (y/n)		0.023	0.852			
ln SD1				8.413	0.267	< 0.001
**HbA1c (%)**	-0.148	-0.323	**0.006**			
**Age (years)**	-0.013	-0.246	**0.035**			
**BMI (kg/m^2^)**	-0.031	-0.233	**0.042**			
Disease duration (years)		-0.036	0.781			
Office blood pressure, systolic (mmHg)		-0.022	0.851			
eGFR (ml/min**/**1.73 m**^2^**)		0.150	0.231			
Use of ACE inhibitors/ARBs (y/n)		0.042	0.752			
ln SD2				10.016	0.372	< 0.001
**HbA1c (%)**	-0.111	-0.296	**0.007**			
**Age (years)**	-0.011	-0.269	**0.014**			
**BMI (kg/m^2^)**	-0.033	-0.302	**0.005**			
**Smoking**	-0.128	-0.219	**0.038**			
Disease duration (years)		0.008	0.949			
Office blood pressure, systolic (mmHg)		0.016	0.885			
eGFR (ml/min**/**1.73 m**^2^**)		0.100	0.388			
Use of ACE inhibitors/ARBs (y/n)		0.062	0.618			
Use of beta receptor antagonists (y/n)		0.054	0.650			

Unstandardized (B) and standardized (β) regression coefficients. Statistically significant p-values (p < 0.05) are formatted in bold.

## Discussion

Serious microvascular and macrovascular complications are frequent in patients with advanced T1DM. Imbalance of the autonomic nervous system is one of the most overlooked of all main complications in this disease (4). Most common symptoms of CAN include resting tachycardia, orthostatic hypotension, poor exercise tolerance, dizziness, lightheadedness, and fragility. These all are consequences of the injury to the autonomic nerve fibers that innervate the heart and blood vessels, resulting in anomalies in heart rate control and vascular tone (4). Various studies have proved that CAN is a risk factor for cardiovascular morbidity and mortality (5, 9, 24, 25).

The DCCT (Diabetes Control and Complications Trial)/EDIC (Epidemiology of Diabetes Interventions and Complications) and further investigations suggested that disease duration and quality of the glycemic control are the main risk factors for CAN (6–9, 26). Nevertheless, major cardiovascular risk factors, such as age, blood pressure, obesity, dyslipidemia, and smoking may also play essential role in the evolution of CAN. In addition, the existence of the diabetic microvascular complications (nephropathy or microalbuminuria and retinopathy) shows strong association with CAN (6–9, 26).

Typical symptoms of CAN present at late stage, whereas early autonomic dysfunction may exhibit no symptoms. HRV, however, is a sensitive biomarker of the autonomic control of the heart. Thus, diminished HRV is considered as the earliest indicator of CAN in T1DM population (3, 4, 10, 11), which has been reported even in young, asymptomatic patients, compared with those in healthy individuals (12–15).

Major risk factors for CAN, especially disease duration and glycemic control, have also been verified as significant determinants of reduced HRV in this disease (13, 16–19). Literature data, however, are inconsistent regarding these questions.

Thus, our work targeted to produce a comprehensive analysis of the HRV in asymptomatic T1DM patients by evaluating time domain, frequency domain, and nonlinear indices. Results were compared with those in healthy subjects. The potential impact of the established CAN risk factors and common cardiovascular risk factors on the HRV parameters was also investigated.

Variability of the consecutive RR intervals reflects the capability of the cardiovascular system to adapt to extrinsic and intrinsic factors. This variability is normally regulated on a beat-to-beat basis by the balance of the sympathetic and parasympathetic nervous systems at the sinus node level (27). SDNN is a measure of overall HRV, reflecting both sympathetic and parasympathetic activation, whereas rMSSD represents the parasympathetic component (13, 28). Total Power represents the overall modulation of the heart rate, whereas HF reflects respiratory-mediated modulation of the heart rate by the parasympathetic system. LF is attributed to the baroreflex-mediated regulation of the heart rate, reflecting both sympathetic and parasympathetic inputs to the sinus node (28, 29). Others view it as a parameter of the sympathetic modulation (28). Thus, LF/HF ratio is considered as an index of sympathovagal balance (13, 29), although this concept has already been challenged (30). VLF is attributed to the modulatory action of the renin-angiotensin system, thermoregulation, and peripheral vasomotor tone (28). SD1 reflects the short-term variability of RR interval variation and is primarily influenced by parasympathetic modulation. SD2, on the other hand, is a parameter of the long-term variability and represents sympathetic activation (31, 32).

In a small group of young T1DM patients, Javorka et al. reported significant reduction in HF band, whereas no significant difference between T1DM group and control population was observed in LF band (14). In the study population of Rosengård-Bärlund et al., T1DM patients showed reduced SDNN, rMSSD, and HF values. LF values remained preserved, resulting in a higher LF/HF ratio (15). In a study on 354 young T1DM individuals, Jaiswal et al. observed significantly lower SDNN and rMSSD values compared with healthy controls. Normalized HF was impaired, but normalized LF was significantly elevated compared with healthy subjects; thus, LF/HF ratio was also elevated (13). Da Silva et al. observed reduction both in sympathetic and parasympathetic activities and in overall variability as matched with the healthy population: SDNN, rMSSD, HF, LF, SD1, and SD2 values were all significantly reduced in their study accompanied by a preserved LF/HF ratio (12). Our findings are in line with the results of da Silva et al. In addition, significantly reduced VLF and Total Power values were found in our population. Regarding the latter two parameters, no previous reports are available in T1DM patients, in comparison with healthy subjects.

Literature data suggest that diminished parasympathetic autonomic tone is detected first in early CAN. As a result, there is a sympathetic predominance, represented by an elevated resting heart rate (25). HF is impaired, but LF power, the surrogate for sympathetic dysfunction, is preserved or even higher in this phase, resulting in a higher LF/HF ratio (13), as it was found in many previous studies (13–15). In more advanced CAN, on the other hand, both the parasympathetic and sympathetic nerves become affected (25), as it is reflected by a balanced LF/HF ratio. This situation was depicted by da Silva et al. (12) and by our study.

In the study of Jaiswal et al., HbA_1c_ remained the independent predictor of SDNN and rMSSD in a young T1DM population in multiple linear regression analysis. The association between HbA_1c_ and LF/HF ratio was not significant. More advanced age and higher triglyceride levels were reported as further independent correlates of lower SDNN and rMSSD. Duration of diabetes, however, was not independently related to any of their HRV indices (16). In another work of Jaiswal et al., older age, female gender, elevated LDL cholesterol and triglyceride levels, and microalbuminuria were all associated with reduced SDNN, rMSSD, HF, and LF but did not account for the observed differences between patients with and without diabetes (13). In addition to SDNN and rMSSD, the standard frequency-domain indices of HRV, including VLF, were also investigated in the work of Pertseva et al, and the diurnal rhythm of the HRV parameters was also taken into consideration (18). In univariable analysis, higher HbA_1c_ had negative effect on HRV, reducing the value of both time domain and frequency domain parameters, except LF/HF ratio. eGFR also showed significant correlation with the same parameters, including LF/HF ratio (18). Logistic regression models, adjusted for gender and age, were used by Pop-Busui et al. to assess the associations between SDNN and HbA_1c_, diabetes duration, systolic blood pressure, as well as any sustained albumin excretion rate (AER) ≥ 30 mg/day in T1DM patients. HbA_1c_ and presence of any sustained AER ≥ 30 mg/day were independently associated with SDNN but only in patients with known CAN (19).

In our study, similarly to the previous findings, no significant correlation was revealed between HbA_1c_ and LF/HF ratio. HbA_1c_, however, was proved to be significant independent predictor of all further time domain, frequency domain, and nonlinear indices, alone, or in combination with common adjustment factors, such as age and BMI. In univariable analysis, eGFR showed significant correlations with various HRV parameters in our study but lost its significance in multiple models, except regarding rMSSD. Serum cholesterol and triglyceride levels did not show any correlation with HRV parameters in our study. In the general population, smoking appeared detrimental on time domain and frequency domain indices of HRV but did not affect LF/HF balance (33). In univariable analysis, smoking showed significant correlation with various HRV parameters in our study but lost its significance in multiple models, except regarding SD2. In hypertensive subjects with or without metabolic syndrome, a complex relationship was reported between HRV, blood pressure, antihypertensive treatment, and the metabolic profile of the patients (34). In our T1DM population, about one-third of the patients were treated with antihypertensive drugs. Office systolic blood pressure and the use of ACE inhibitors/ARBs/beta receptor antagonists showed various correlations with the HRV parameters, except with LF/HF ratio. These correlations, however, lost their significance in multiple models.

Although, in T2DM patients, female gender seems to be a risk factor for CAN (25), in T1DM population, EURODIAB IDDM Complications Study found no significant difference in the prevalence of CAN between male (35%) and female (37%) patients (7). Similarly, gender did not influence the HRV parameters in our study.

Duration of diabetes remained the only independent predictor of LF/HF ratio in our study: Longer disease duration is associated with the increase of LF/HF ratio. This may represent the progression of the sympathetic predominance (vagal withdrawal) during the course of the disease (11). In addition, recent literature suggests that higher LF/HF ratio is more common in T1DM patients with frequent hypoglycemic episodes (17, 35) and the frequency of hypoglycemic episodes increasing with longer disease duration (17).

Our results suggest that HRV parameters calculated from a 5-min segment of the ECG recording are reliable and allow early detection of the first, subclinical stage of CAN in asymptomatic T1DM population. This may provide more time to vigorously manage diabetes and may increase the efficacy of interventions in order to prevent manifest CAN.

We are aware of many limitations of our study. First, the size of our study population was limited by reason of the demanding selection of asymptomatic patients without cardiovascular comorbidities. Treadmill stress test was used for screening subjects suspicious for CAD. Due to ethical reasons, the use of coronary angiography was avoided in the asymptomatic patients and volunteers. The data about the hypoglycemic episodes of our T1DM patients are not available. Non-pharmacological treatment options—dietary intervention, regular exercise (aerobic and/or resistance training), weight loss and psychological counseling—have been reported to be useful in preventing CAN or mitigating its symptoms (36). Data about these factors, however, were not recorded in our study. Usual cardiovascular medication of the patients was not withdrawn before testing. Nevertheless, correlations between HRV parameters and the use of ACE inhibitors/ARBs/beta receptor antagonists lost their significance in multiple models. Lipid-lowering medication use of the patients was not considered. Healthy subjects with positive smoking history or with known dyslipidemia were not excluded from the study. Smoking habits did not differ between the two populations. Lipid levels of the healthy population, however, were neglected.

## Conclusion

Our data confirm that asymptomatic T1DM patients have significantly reduced overall HRV as compared with healthy subjects, indicating early, subclinical CAN.

Quality of the glycemic control is an important determinant of HRV among T1DM patients. This relationship is independent of other risk factors for CAN or traditional cardiovascular risk factors and underlines the central role of tight glycemic control in the modulation of cardiac autonomic function in T1DM population.

## Data availability statement

The raw data supporting the conclusions of this article will be made available by the authors, without undue reservation.

## Ethics statement

The studies involving humans were approved by Regional Research Ethics Committee, Clinical Centre, University of Pécs. The studies were conducted in accordance with the local legislation and institutional requirements. The participants provided their written informed consent to participate in this study.

## Author contributions

RF, NV-V, LH, and IW designed the study. MH, VV, and GM carried out subject recruitment. RF performed echocardiography. MH and KG collected ECG data and performed HRV analysis. MH and RF analyzed and interpreted the data and drafted the manuscript. KG, VV, and NV-V helped in literature analysis, contributed to discussion of the results. GM, IW, and LH reviewed the manuscript. All authors contributed to the article and approved the submitted version.
